# Global Methylation and Protamine Deficiency in Ram Spermatozoa Correlate with Sperm Production and Quality but Are Not Influenced by Melatonin or Season

**DOI:** 10.3390/ani10122302

**Published:** 2020-12-04

**Authors:** Kelsey R. Pool, Jessica P. Rickard, Simon P. de Graaf

**Affiliations:** Faculty of Science, School of Life and Environmental Sciences, The University of Sydney, Sydney, NSW 2006, Australia; jessica.rickard@sydney.edu.au (J.P.R.); simon.degraaf@sydney.edu.au (S.P.d.G.)

**Keywords:** melatonin, sperm, protamine, methylation, reproductive seasonality, testicular function

## Abstract

**Simple Summary:**

Though environmental factors can alter the epigenome of mammalian spermatozoa, it is currently unclear whether these epigenetic changes are linked to sperm production, quality and fertility. This study aimed to identify whether the hormone melatonin, responsible for upregulating ram reproductive function, is able to alter broad epigenetic markers in spermatozoa, namely sperm global methylation and protamine deficiency. It was also investigated whether these parameters corresponded to ram endocrinology, semen production and quality. Though no effects of season or melatonin were found, both sperm global methylation and protamine deficiency correlated with several semen production and quality parameters. These moderate associations with sperm production and quality support that sperm protamine deficiency and global methylation are broadly indicative of testicular function.

**Abstract:**

This study assessed whether the seasonal effects of melatonin that upregulate ram reproductive function alter sperm global methylation or protamine deficiency and whether these parameters corresponded to ram endocrinology, semen production and quality. Ejaculates were assessed from rams that received melatonin implants (*n* = 9) or no implants (*n* = 9) during the non-breeding season. Ejaculates (*n* = 2/ram/week) were collected prior to implantation (week 0), 1, 6 and 12 weeks post implantation and during the following breeding season (week 30). Flow cytometry was used to assess the sperm global methylation and protamine deficiency in each ejaculate, which had known values for sperm concentration, motility, morphology, DNA fragmentation, seminal plasma levels of melatonin, anti-Mullerian hormone and inhibin A. Serum levels of testosterone and melatonin were also evaluated. Though there was no effect of melatonin or season, sperm protamine deficiency was negatively correlated with sperm production and seminal plasma levels of anti-Mullerian hormone and positively correlated with sperm DNA fragmentation and morphology. Global methylation of spermatozoa was positively correlated with sperm DNA fragmentation, morphology and serum testosterone and negatively correlated with sperm motility. These moderate associations with sperm production and quality suggest that sperm protamine deficiency and global methylation are indicative of ram testicular function.

## 1. Introduction

Environmentally induced epigenetic changes in the male gamete are of increasing interest, as they emerge to be key drivers of developmental plasticity across generations [1]. Established predominantly in humans and mice, exposure of males to factors such as diet, exercise, pollutants and other environmental cues results in marked changes to the epigenome of spermatozoa [2,3,4,5], though the relationship between such changes and sperm fertility is yet to be fully elucidated. These effects upon the sperm epigenome are explored to a far lesser extent in domestic livestock, despite the exposure of males to a range of environmental conditions, stressors and diets in commercial production systems. Of particular interest, seasonal breeders, such as sheep, are dependent upon environmental cues for successful reproduction [6,7] and as such undergo marked seasonal changes. In spite of these fluctuations in reproductive capacity, there is little investigation upon differing epigenetic patterns in ram spermatozoa in response to season. 

Ovine reproductive seasonality is primarily regulated by the neurohormone melatonin, secreted in response to photoperiod exposure [8,9,10]. Whilst ewes entirely cease ovarian activity during the non-breeding season, rams continue to produce spermatozoa [11,12], thus exposing viable sperm cells to a range of differing environmental, and physiological, conditions. Though it is well established that most ram breeds exhibit a reduction in testicular size and sperm production in response to the environmental changes delineating the non-breeding season [11,12], there are many discrepancies concerning seasonal changes in sperm function and fertilising capacity [12,13,14]. There is thus considerable ambiguity surrounding the practice of collecting semen for artificial insemination during the non-breeding season, as despite negative perceptions, it is unclear if sperm function is truly reduced during this period. Furthermore, though it appears evident that ram testicular function is able to be improved utilising slow-release melatonin implants [8,15,16,17], thereby increasing sperm production, there are conflicting reports regarding whether melatonin exposure is able to seasonally modulate sperm functionality [8,16,18,19]. Regardless of whether reproductive upregulation in the ram occurs naturally or is artificially stimulated, spermatozoa are exposed throughout the spermatogenic cycle to the different endocrine conditions stimulated by melatonin, such as upregulated testosterone production at both a systemic and a testicular level [15,20], as well as to melatonin itself. Given that melatonin is shown to induce functional changes to spermatozoa in an in vitro setting [21,22,23], and possibly when used exogenously [16], this brings into question as to whether spermatozoa exposed to breeding season conditions have altered function and fertility and if this may be reflected in broad changes to the sperm epigenome.

Broad epigenetic changes within spermatozoa are now known to occur in response to external factors, the resulting epigenetic profiles emerging as being vital to reproductive success [24,25,26]. One primary mechanism of interest, DNA methylation, is reported to influence sperm development at several stages of the spermatogenic cycle [27,28,29]. A constantly dynamic process where a methyl group is added to cytosine in CpG dinucleotides, methylation regulates various cell processes and gene expression [1,30]. This reversible epigenetic programming appears to be impacted by several factors local to the testes, including testicular pathologies and endocrine fluctuations. Accordingly, the level of global DNA methylation in human [31,32], boar [33] and rat [34,35] spermatozoa is associated with sperm quality. Cumulatively, such studies suggest that global methylation may be a valuable marker of sperm competence and one that could plausibly be affected by the seasonal and environmental changes encountered in animal production systems. 

The DNA of spermatozoa is similarly subjected to external environmental cues and stressors [36]. The corresponding degree of damage appears to be somewhat conditional to the integrity of sperm chromatin, the structural packaging of which is dependent upon protamines [37,38]. Protamines, the primary nuclear proteins of sperm cells, are responsible for packaging sperm chromatin into a semi-crystalline state [37,38]. Mouse models with decreased quantities of protamine proteins demonstrate abnormal sperm morphology, abnormal chromatin packaging, DNA damage and male infertility [39], indicating that sperm protamine deficiency can have detrimental impacts upon male fertility. Though studies in some domestic livestock species support this theory [33,40], there is no investigation of this relationship in the ram, nor any evidence of changes in response to the seasonal upregulation of the reproductive function. 

Whether broad epigenetic changes in spermatozoa occur in response to seasonal cues remains to be elucidated. Accordingly, as sheep have a known mechanism for regulating seasonal reproduction, this study used the ram to investigate whether reproductive seasonality alters global methylation or protamine deficiency in spermatozoa. Here, we investigated whether any alterations existed between the natural breeding and non-breeding periods or if changes were able to be induced by artificially mimicking the onset of the breeding period using slow-release melatonin implants during the non-breeding season. Furthermore, to support the hypothesis that epigenetic changes occur in response to the seasonal upregulation of the reproductive function, we aimed to identify any relationships between sperm global methylation, protamine deficiency and ram endocrinology, semen production and quality.

## 2. Materials and Methods 

### 2.1. Animals 

This study was carried out in strict accordance with the Australian Research Act 1985 No. 123 and the Australian code for the care and use of animals for scientific purposes 8th edition (2013). All experimental procedures were conducted with approval from the University of Sydney Animal Ethics Committee (approval 2017/1155). 

All animals were housed at the University of Sydney Sheep Research Unit, Cobbitty NSW, Australia. Poll Dorset (*n* = 9) and Merino (*n* = 9) rams were maintained as a single mob on pasture. Rams had access to improved pasture (*Elymus repens*, *Pennisetum clandestinum*, *Themeda triandra*, *Lolium*, *Cytisus scoparius*, *Arrhenatherum elatius*, *Triticosecale*) and water ad libitum. Additional lucerne and lupins were supplemented up to three times a week. All rams were approximately 2 years of age, considered of standard reproductive health (testicular size within normal range, no lesions, no morphological abnormalities of the external reproductive anatomy) and within a normal body condition score range (between 3 and 4) throughout the study period.

### 2.2. Experimental Design

Following an initial two weeks of data collection during the non-breeding season (September), approximately half the rams in each breed were randomly allocated to a treatment group, where they either received 3 × 18 mg melatonin implants (Regulin, CEVA Animal Health, Australia, Merino = 5, Poll Dorset = 4) or underwent the same implantation process with empty implanter guns to act as a control group (Merino = 5, Poll Dorset = 4). Treatment groups were balanced for animal weight.

Two ejaculates were collected from each ram once per week throughout the non-breeding season (September–December) and in the following breeding season (April). Samples selected for inclusion in the present study were chosen retrospectively following the completion of a previous study [15]. The study weeks selected for inclusion were week 0 (prior to implantation), week 1 (1 week post implantation), week 6, week 12 and week 30 (the following breeding season). For correlations between sperm protamine deficiency and global methylation and ejaculate quality, the ejaculate (total *n* = 180) was considered the experimental unit.

### 2.3. Semen Collection and Preparation

Ejaculates were collected from rams using an artificial vagina in the presence of a teaser ewe. For correlation purposes, the following parameters for each ejaculate were established prior to the present study [15]: melatonin concentration in seminal plasma, anti-Mullerian hormone (AMH) concentration in seminal plasma, inhibin A concentration in seminal plasma, ejaculate concentration, ejaculate volume, the number of spermatozoa per ejaculate, sperm motility, the percentage of abnormal spermatozoa and sperm DNA fragmentation. The concentration of testosterone and melatonin in blood serum was also obtained during the same study weeks as ejaculate collections.

Spermatozoa were isolated from seminal plasma by centrifugation of the whole ejaculate at 14,000 g for 10 min and removal of the supernatant. This process was undertaken twice to ensure seminal plasma was absent from the pelleted sperm cells. Spermatozoa were snap-frozen and stored in liquid nitrogen until analysis.

### 2.4. Chemicals

Unless otherwise stated, all chemicals were sourced from Sigma- Aldrich (St Louis, MO, USA). Anti-5-methylcytosine (5-mC) antibody and donkey anti-mouse IgG H&L (FITC) were purchased from Abcam (Abcam Australia Pty Ltd., Melbourne, Victoria, Australia), and propidium iodide (PI) was sourced from Invitrogen (Thornton, NSW, Australia).

### 2.5. Flow Cytometry

Spermatozoa were assessed using a CytoFLEX flow cytometer (Beckman Coulter, Lane Cove, Australia). Sperm cells were isolated from total events based on forward and side-scatter profiles. The concentration of spermatozoa in the assessed samples was between 2 and 5 × 10^6^ spermatozoa/mL, and a total of 10,000 spermatozoa per sample were recorded. Samples were analysed using CytExpert 2.0 software (Beckman Coulter, Lane Cove, Australia). 

#### 2.5.1. Protamine Deficiency

The samples were assessed for protamine deficiency as previously described [33,41], with some adjustments. Snap-frozen samples were thawed on ice, and an aliquot diluted to approximately 2 × 10^6^ sperm cells in 200 µL of 1 × TNE buffer (0.15 M NaCl, 0.01 M Tris HCl, 1 mM disodium EDTA pH 7.4). The samples were then washed once in 1 × TNE (300 g for 10 min) and resuspended in 100 µL of McIlvaine’s buffer (17 mL 0.1 mol/L citric acid mixed with 83 mL 0.2 mol/L Na_2_HPO_4_ and 10 mmol/L MgCl_2_, pH 7.0) containing 0.25 mg/mL chromomycin (CMA_3_). The cells were stained for 20 min and then counterstained with PI at a final concentration of 6 µM for 10 min to assist with the gating of the sperm population.

CMA_3_ fluorescence from the gated cells was obtained through a 528/45 bandpass filter after excitation with a violet laser (405 nm). The degree of protamine deficiency was assessed by recording the median CMA_3_ fluorescence of the sperm population.

#### 2.5.2. Global Methylation

The samples were assessed for global methylation as previously described [42,43], with some adjustments. Snap-frozen samples were thawed on ice, and an aliquot adjusted to approximately 5 × 10^6^ sperm cells in 200 µL of 1 × TNE buffer (0.15 M NaCl, 0.01 M Tris HCl, 1 mM disodium EDTA pH 7.4). All following steps were performed at room temperature.

To decondense sperm DNA, the samples were incubated for 20 min in 1 mol/L hydrochloric acid (HCl)–Tris buffer, pH 9.5, containing 25 mmol/L dithiothreitol. The samples were then washed twice (300 g for 10 min) in phosphate-buffered saline containing 0.5% Tween (PBS-T 0.5%). Spermatozoa were then incubated with HCl (6 N) for 15 min to denature sperm DNA. The samples were again washed twice, firstly with a Tris solution (1 mol/L, pH 9) and then in PBS-T 0.5%. Following this, the samples were incubated with the mouse anti-5-mC antibody at 1 µg/mL in PBS-T 0.5% for 20 min. The samples were washed twice with PBS-T 0.5% to remove any excess antibody. The washed samples were then incubated with anti-mouse antibodies conjugated with fluorescein isothiocyanate (FITC) for 20 min before being counterstained with PI at a final concentration of 6 µM for 10 min, to assist with the gating of the sperm population.

Green fluorescence, representing the methylation level, was obtained through a 533/30 nm band-pass filter after excitation with a blue (488 nm) laser. The level of methylation was determined by recording the median FITC fluorescence of the sperm population.

### 2.6. Statistical Analysis

All statistical analysis was undertaken using Genstat (version 18, VSN International).

Correlations between global methylation, protamine deficiency and all other parameters were investigated by assessing associations using linear regression. If significant (*p* < 0.05), the Spearman nonparametric correlation test was then used to determine correlations between variables, using two-tailed *p* values. Correlations were considered significant if *p* < 0.05.

To determine any effect of the melatonin treatment, linear mixed-model regression (REML) was used. For the layout of these models, study week, treatment and breed were set as fixed effects, and ram, breed and ejaculate were set as random effects. Normality and homogeneity of residual variances were confirmed using the Shapiro–Wilk test and the Bartlett’s test, respectively.

## 3. Results

### 3.1. Protamine Deficiency

**Figure 1:**
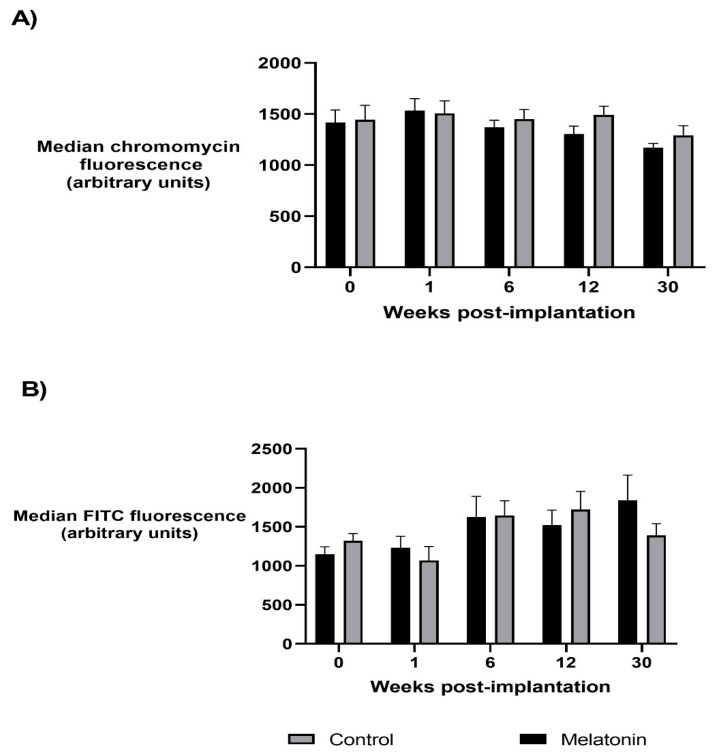
The relative levels of sperm protamine deficiency (**A**) and global methylation of the sperm epigenome (**B**) in ejaculates collected from melatonin-treated and control rams over the study period. Changes in protamine deficiency (**A**) and global methylation (**B**) are represented by the median chromomycin and FITC fluorescence of the sperm population, respectively. Values are the mean ± SEM; no significant difference is reported between treatment groups or study weeks (*p* > 0.05).

As shown in Figure 1, linear mixed models demonstrated no significant difference in sperm protamine deficiency between melatonin and control treatment groups (*p* > 0.05) or study weeks, though in study week 30 (breeding season), protamine deficiency tended to be lower compared to the non-breeding season weeks for all rams, regardless of treatment group (*p* = 0.074). Sperm protamine deficiency was not correlated with levels of melatonin in the seminal plasma or blood (*p* > 0.05).

**Figure 2:**
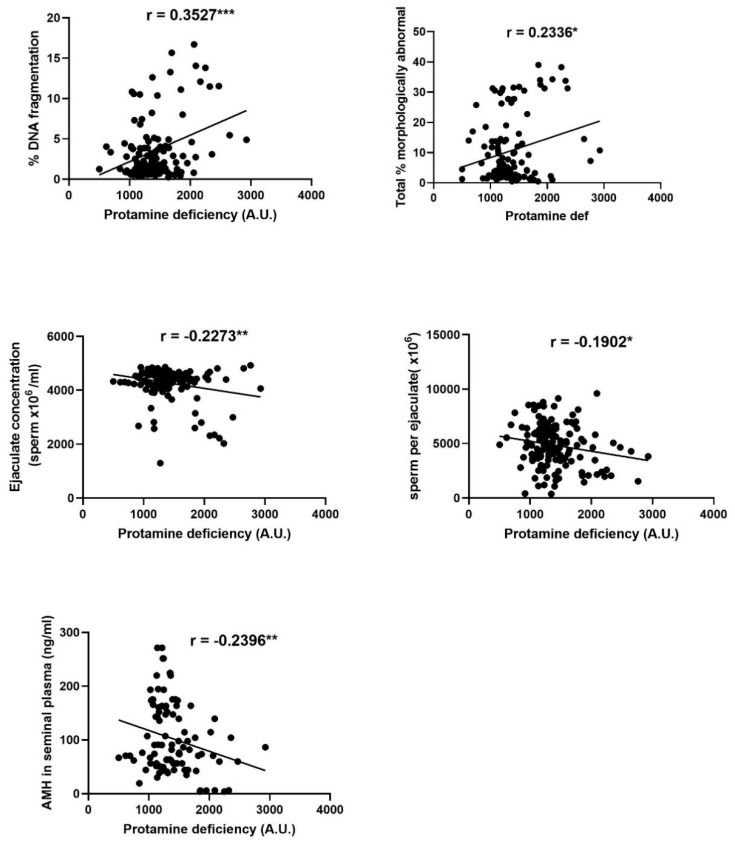
Significant correlations between sperm protamine deficiency and sperm DNA fragmentation, sperm concentration per ejaculate, the number of spermatozoa per ejaculate, anti-Mullerian hormone (AMH) in seminal plasma and the percentage of abnormal spermatozoa. Each data point plotted represents an individual ejaculate. Asterisks indicate the significance of the correlation * *p* ≤ 0.05, ** *p* ≤ 0.01, *** *p* ≤ 0.001.

Significant relationships were observed between sperm protamine deficiency and sperm DNA fragmentation, the percentage of morphologically abnormal spermatozoa, the concentration and number of spermatozoa per ejaculate and the level of AMH in seminal plasma, as summarised in Figure 2.

### 3.2. Sperm Global Methylation

Linear mixed models demonstrated no significant difference in the levels of sperm global methylation between treatment groups or study weeks (*p* > 0.05), nor was sperm global methylation correlated with the levels of melatonin in the seminal plasma or blood (*p* > 0.05).

**Figure 3:**
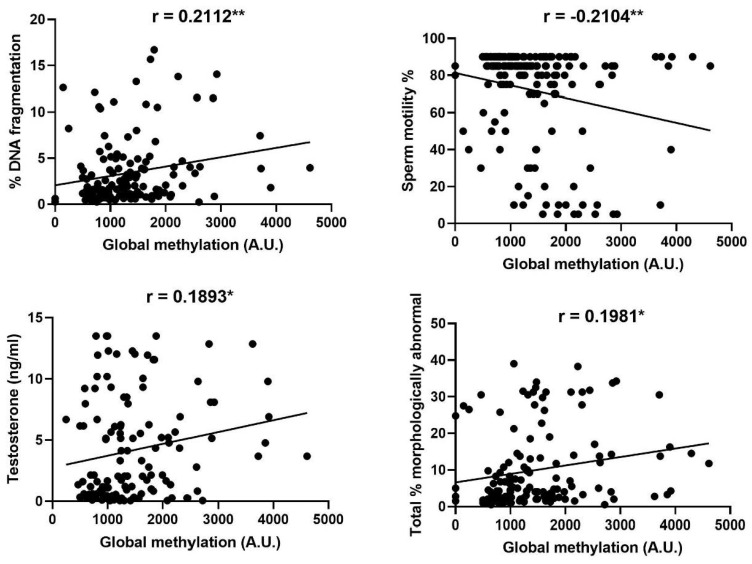
Significant correlations between levels of global methylation in spermatozoa and sperm DNA fragmentation, sperm motility, morphology and the concentration of testosterone in blood serum. Each data point plotted represents an individual ejaculate. Asterisks indicate the significance of the correlation * *p* ≤ 0.05, ** *p* ≤ 0.01.

Significant relationships were observed between levels of global methylation and sperm DNA fragmentation, sperm motility and morphology, and the concentration of testosterone in blood serum, as summarised in Figure 3.

## 4. Discussion

To our knowledge, this is the first study in the ram to characterise relationships between sperm global methylation, protamine deficiency, ram reproductive endocrinology and sperm production and quality. Though some of these correlations have been demonstrated in other species, namely humans [44,45,46], we show that relationships between epigenetic modifications and semen parameters similarly exist in the ram. Given that epigenetic variations in spermatozoa are now known to occur in response to environmental factors, including changes in nutrition, climate and other external cues [1], we hypothesised that both exogenous melatonin and season would influence the global methylation and protamine status of ram spermatozoa. However, we found no effect of either melatonin or season, despite previously observing differences in ram testicular function in response to melatonin treatment [15]. As the correlations found in the current study were generally only moderately associated, this may account for why melatonin-induced changes to semen parameters were not reflected in patterns of either sperm global methylation or protamine status. Regardless, this study suggests the function of ram spermatozoa is not inferior during the non-breeding period, nor alterations to the broad epigenome are conferred.

In line with previous literature, we found the degree of DNA fragmentation in ram spermatozoa to be positively correlated with both sperm global methylation and protamine deficiency. Given that protamines are an essential part of the sperm DNA packaging process [37,38], the latter result is not unsurprising, this relationship in human spermatozoa having been described in a previous meta-analysis [46]. Though it would appear logical that increased DNA fragmentation corresponds to protamine deficiency, the nature of the relationship between these two parameters is far from established. Protamines are ideally present in spermatozoa in a species-specific ratio of protamines 1 and 2, where the replacement of histones with protamines occurs throughout spermatogenesis [47]. Irregular protamine ratios are related to both sperm DNA damage and male sub-fertility [46,48], indicating that protamine status is likely essential for normal embryonic development. Interestingly, though spermatozoa with damaged DNA can display as morphometrically normal and have been reported to maintain fertilisation capacity, this damage may later translate to impaired embryo development following fertilisation [49]. In the present study, the positive correlation between sperm protamine deficiency and DNA fragmentation, but the lack of relationship with sperm functionality, indicates this may indeed represent an issue in the ram. From an industry perspective, spermatozoa that appear functionally normal and are thus used for insemination, only to result in poor embryo development, allow sub-fertile sires to persist in breeding programs, perpetuating poor reproductive efficiency. It is interesting to note that the increase of melatonin, a potent antioxidant [50], in seminal plasma did not decrease the degree of sperm protamine deficiency, as it is theorised that both DNA fragmentation and protamine deficiency are the result of excessive levels of reactive oxygen species (ROS) [46]. Even within our limited sample size, we did observe a trend (*p* = 0.074) describing reduced protamine deficiency during the breeding season. This warrants further study in the ram, as it is currently unclear if and how there are seasonal advantages conferred to mammalian sperm DNA [51,52], though there is some evidence that this occurs in mussels [53].

Though levels of protamine deficiency were not related to sperm function, we did observe correlations to sperm production and morphology, with ejaculates containing lower numbers of spermatozoa and higher percentages of morphologically abnormal spermatozoa having a greater degree of protamine deficiency. Whilst treatment of rams with exogenous melatonin increased sperm production in our previous study [15], we did not similarly see an effect of treatment upon sperm protamine deficiency, possibly due to the assessment of a subset of study weeks in the present study. Regardless, the correlation between sperm protamine deficiency, ejaculate concentration and sperm morphology has been similarly observed in humans [44,45], though to our knowledge this is the first study to suggest that these relationships also exist in the ram. Given that our previous study in the ram established a positive correlation between the number of spermatozoa per ejaculate and AMH concentration in seminal plasma [15], it is interesting to note that seminal plasma levels of AMH comparably relate to sperm protamine deficiency. In other species, AMH is an emerging indicator of testicular function, where seminal plasma AMH levels positively correlate with elevated spermatogenesis [54,55], reduced oxidative stress [54,56] and increased semen production [57] and quality [58,59,60,61,62]. In the present study, lower levels of seminal plasma AMH were associated with increased protamine deficiency, supporting the theory that AMH levels in seminal plasma may be somewhat predictive of testicular function.

The methylation of DNA is becoming increasingly investigated in a reproductive context, emerging to have a much more prominent role in sperm functionality than previously assumed. The present study revealed that global methylation in ram spermatozoa is correlated with sperm quality, with ejaculates with lower sperm motility and higher percentages of morphologically abnormal spermatozoa demonstrating greater levels of methylation. There are now several studies suggesting that both patterns and degree of methylation vary between highly and lowly motile sperm populations in the bull [63], normal and sub-fertile men [64,65,66] and high- and low-fertility buffalo [67]. The correlation between sperm global methylation and semen parameters is less explored in comparison to the relationship between methylation and fertility, with limited previous findings yet to definitively conclude the nature of the association between global methylation and semen parameters. In comparison with the present study, some investigations in human spermatozoa agree that overall hypermethylation is associated with ejaculates of lower motility [68], though others indicate the contrary [65]. As these studies assess human spermatozoa, it cannot be concluded if such discrepancies are due to a species-specific difference, variation in the cause of sub-fertility or the different methodologies used to quantify methylation. Regardless of whether sperm populations demonstrate extreme hyper- or hypomethylation, there appears to consistently be a relationship with motility, suggesting that spermatozoa on either end of the methylation spectrum demonstrate impaired motility. Given that sperm motility is a key characteristic used to determine ejaculate fertility in the sheep production industry [69,70], external factors that alter motility are of interest. In this case, we cannot conclude upon any causation behind the relationship between global methylation and sperm motility but can speculate that this may partially be attributed to the other correlations revealed in this study. Inferior quality semen samples are likely to present poor values of the majority of the observed parameters; for example, reduced sperm DNA integrity is often accompanied by lower degrees of motility and normal morphology [71,72,73]. Here, we similarly saw that methylation was higher in samples with a greater degree of sperm DNA fragmentation and abnormal morphology, and as such the hypermethylation of ram spermatozoa may be related more broadly to poorer quality ejaculates, possibly reflecting altered testicular function, rather than specifically to low sperm motility.

As we did not see any influence of melatonin upon motility in our previous study, it is perhaps unsurprising that neither melatonin concentration in the seminal plasma nor season influenced the global methylation of ram spermatozoa. Curiously, and somewhat contrary to this, we did find a positive correlation between ram serum testosterone and sperm global methylation, though testosterone was increased as a result of melatonin treatment in the previous study [15]. As we found increasing levels of methylation to be mainly associated with poorer semen quality, this result is somewhat unexpected, as greater levels of testosterone have previously been related to upregulated testicular function [74,75]. However, it is possible that external factors, such as heat or other stressors, could rapidly influence semen quality and potentially alter sperm DNA methylation, without exerting an effect on testosterone production. This relationship, which was relatively weak despite its statistical significance in the current study, should be clarified under more controlled conditions.

## 5. Conclusions

To conclude, this study found no evidence that either melatonin or season directly exerts an effect upon the global methylation or protamine status of ram spermatozoa. This indicates that, at a broad, non-specific level at least, the epigenome of ram spermatozoa is likely not compromised during the non-breeding season and challenges the negative perceptions surrounding semen collection during this period. However, we did observe relationships between the global methylation and protamine deficiency and ram endocrinology, sperm quality and production, which appear to be consistent with findings in other species. Specifically, the observed associations between protamine deficiency, sperm production, morphology, sperm DNA fragmentation and AMH concentration in seminal plasma suggest that sperm protamine deficiency may indicate testicular function. Similarly, our findings that sperm global methylation is linked to sperm motility, morphology and DNA fragmentation indicate that epigenetics may hold a role in ram sperm functionality, as similarly indicated in humans [65,68], cattle [63,67] and pigs [33]. Our observed correlations warrant further study in the ram in order to clarify the influence of external factors and environmental cues on global methylation and protamine status and to determine any subsequent effects upon fertility.
